# SIRT4 silencing in tumor-associated macrophages promotes HCC development via PPARδ signalling-mediated alternative activation of macrophages

**DOI:** 10.1186/s13046-019-1456-9

**Published:** 2019-11-19

**Authors:** Zhi Li, He Li, Zhi-Bo Zhao, Wei Zhu, Pan-Pan Feng, Xi-Wen Zhu, Jian-Ping Gong

**Affiliations:** 1 Department of Hepatobiliary Surgery, the Second Affiliated Hospital of Chongqing Medical University, Yuzhong District, Chongqing, 400010 People’s Republic of China; 2 Department of General Surgery, the YongChuan Hospital of Chongqing Medical University, YongChuan District, Chongqing, 402160 People’s Republic of China

**Keywords:** Sirtuin 4, HCC, Tumour-associated macrophages, NF-ΚB, PPARδ/STAT3, Inflammatory cytokines

## Abstract

**Background:**

The activation of tumour-associated macrophages (TAMs) contributes to the progression of hepatocellular carcinoma (HCC). SIRT4 acts as a tumour suppressor of tumour growth by regulating cell metabolism, inflammation, and anti-tumourigenesis. However, the involvement of SIRT4 in the activation of TAMs is unknown. Based on previous findings, the expression of SIRT4 in distinct groups of TAMs as well as the effect of SIRT4 silencing on macrophage polarization was investigated.

**Methods:**

The expression of SIRT4 in HCC tissues and peritumour tissues was tested by qRT-PCR, western blotting and histological analysis. A Kaplan-Meier survival curve was generated based on the expression of SIRT4 in the HCC samples. Next, immunofluorescence staining was used to evaluate distinct groups of TAMs in human HCC samples, and the expression of SIRT4 in M1 and M2 TAMs was examined by flow cytometry. A homograft mouse model was used to assess the effect of SIRT4 silencing in TAMs on the development of HCC cells.

**Results:**

SIRT4 was significantly downregulated in HCC tumour tissues, and the expression of SIRT4 in peritumour tissues was positively associated with survival in patients. We further found that downregulation of SIRT4 was associated with increased macrophage infiltration and a high ratio of M2/M1 macrophages in HCC peritumour tissues. Using gene interference, we found that SIRT4 silencing in TAMs significantly modulated the alternative activation of macrophages and promoted in vitro and in vivo HCC cell growth. Mechanistically, we revealed that HCM restricted the expression of SIRT4 in macrophages and promoted alternative activation of macrophages via the FAO-PPARδ-STAT3 axis. Furthermore, we also revealed that elevated MCP-1 expression induced by SIRT4 downregulation was responsible for increased TAM infiltration in peritumour tissues.

**Conclusions:**

Overall, our results demonstrate that downregulation of SIRT4 in TAMs modulates the alternative activation of macrophages and promotes HCC development via the FAO-PPARδ-STAT3 axis. These results could provide a new therapeutic target for the treatment of HCC.

## Background

Hepatocellular carcinoma (HCC) is among the top causes of cancer-related mortality [1]. While great strides have been made in treating HCC, the most common therapies remain surgical resection and liver transplantation. Unfortunately, the high mortality of liver cancer is related to its high recurrence and metastasis rate [2, 3]. As these outcomes mostly occur in the postoperative residual liver, recent studies have highlighted the significance of the tumour microenvironment in the development, metastasis, and recurrence of HCC [4, 5]. Tumour-associated macrophages (TAMs) are copious in the tumour microenvironment and vital in tumour development and metastasis [6]. TAMs usually polarise to the M2-like phenotype [7, 8] and express high levels of IL-10, CD206, and arginase (Arg)-1, while producing low levels of inducible nitric oxide synthase (iNOS), IL-12, and tumour necrosis factor-ɑ (TNF-ɑ).

SIRT4 is a member of the Sirtuin family (SIRT1–7) that affects cellular proliferation, stress resistance, metabolism regulation, inflammation and cancer [9]. SIRT4 performs the role of an ADP-ribosyltransferase, exhibiting demalonylase and deacetylase behaviours in certain tissues [10]. As a mitochondrial sirtuin, SIRT4 is involved in fatty acid oxidation as well as mitochondrial gene expression in liver and muscles [11]. In addition, SIRT4 can inactivate glutamate dehydrogenase to inhibit tumour formation [12]. Recent studies have found that Sirt4 can affect the inflammatory response in several tissues. It has been reported that SIRT4 suppresses pro-inflammatory cytokines in human umbilical vein endothelial cells [13, 14], and some studies have reported that SIRT4 plays an important role in resolving immune tolerance in monocytes [15]. However, no evidence currently articulates the effect of SIRT4 on the inflammatory response in the liver.

The results of this study demonstrate that downregulation of SIRT4 in TAMs and para-cancerous hepatocytes affects the development of HCC as well as the prognosis of HCC patients. In this study, we found that downregulation of SIRT4 in TAMs modulates the alternative activation of macrophages via the FAO-PPARδ-STAT3 axis and that downregulation of SIRT4 in para-cancerous hepatocytes promoted macrophage infiltration by enhanced MCP-1 expression via the NF-κB pathway. Therefore, SIRT4 is a promising target in HCC immunotherapy and reverses macrophage-induced immunosuppression in the tumour microenvironment.

## Materials and methods

### Cell lines and cell cultures

Human HCC cell lines (Huh7 and HepG2) and mouse hepatoma cell lines (H22 and Hepa1–6) were purchased from American Type Culture Collection (ATCC, Rockville, MD, USA). These cell lines were preserved in Dulbecco’s modified Eagle’s medium (DMEM; HyClone, Logan, UT, USA) plus 10% foetal bovine serum (FBS) and 1% penicillin G and streptomycin at 37 °C in humidified air containing 5% CO2. The human monocytic cell line THP-1 was cultured in 1640 supplemented with 10% foetal calf serum.

### Human subjects

A tissue microarray that included 90 HCC tissues and matched surrounding tissues collected between 2007 and 2009 was purchased from Shanghai Outdo Biotech (Shanghai, China). The gender, age, stage, tumour size, pathological type, and clinical stage of patients were also obtained (Additional file 1: Table S1). All patients were followed for 4–6 years. Fresh HCC tissues and matched surrounding tissues were obtained from primary surgery patients interned at the Second Affiliated Hospital Surgery Department of Chongqing Medical University. No chemotherapy or radiotherapy was allowed before surgical treatment. Pathologists evaluated all samples for histological diagnosis. All patients involved in this study provided informed consent that their tissues could be retained and analysed for research purposes only. The Human Research Ethics Committee of the Second Affiliated Hospital of Chongqing Medical University approved this study.

#### Immunohistochemistry

Immunohistochemical staining was carried out according to a prior protocol. The staining intensity was scored as follows: 0, no staining; 1, weak staining; 2, intermediate staining; and 3, strong staining. The positive rate score was determined as follows: 0, 0 of the cells stained positive; 1, 1–20% of the cells stained positive; 2, 21–40% of the cells stained positive; 3, 41–60% of the cells stained positive; 4, 61–80% of the cells stained positive; and 5, 81–100% of the cells stained positive. The total score was the combination of the staining intensity and positive staining rate scores. Samples with a total score <6 and ≥ 6 were defined as the low and high expression groups, respectively.

#### In vivo tumourigenicity

Our institutional ethical board for animal experiments approved all experimental procedures, which followed the Guide for the Care and Use of Laboratory Animals. Four-week-old BALB/c nude mice (*n* = 30) were kept in a sterile environment to serve as hosts to the HCC homografts. H22 cells together with homologous peritoneal SIRT4-knockdown macrophages (PMs) at a ratio of 6:1 were injected subcutaneously into the liver of BALB/c mice (*n* = 30) to prepare the homografts. Five days after inoculation, three mice were sacrificed every 3 days, and the weight and volume of the extracted tumours were calculated. The tumours were dissected approximately 2 mm from the liver tumour margins.

### Lentivirus-mediated overexpression or knockdown of SIRT4

The lentivirus-based SIRT4 overexpression or knockdown vector was constructed according to a prior protocol [12]. Lentivirus packaging and cell transduction were also carried out according to a prior protocol [16].

### HCC-conditioned medium

H22 cells were cultured in serum-free DMEM for 24 h. The supernatants were collected as HCC-conditioned medium (HCM). PMs were cultured with different amounts of HCM or different exposure times in 6-well plates. Different amount of HCM were mixed with complete medium to reach a volume of 2000 μl and percentages of 0% (0 μl), 5% (100 μl), 10% (200 μl), 15% (300 μl), and 20% (400 μl).

### Cell apoptosis assay and flow cytometry measurements

M1-like TAMs were harvested after co-culture with the supernatant of SIRT4-knockdown M2-like TAMs. Then, Annexin V-FITC/PI Cell Apoptosis kit (KeyGen, Nanjing, Jiangsu, China) was used to perform an apoptosis assay. To complete this assay, a suspension (100 μl) of 5 × 10^5^ TAMs were incubated at room temperature with 5 μl of Annexin V and 1 μl of propidium iodide (PI) for 15 min. Flow cytometry (BD Pharmingen, San Diego, CA, USA) was used to measure the apoptotic rate.

### Oxygen consumption

Oxygen consumption rates (OCR) were measured in XF assay media under basal conditions(unbuffered XF assay medium containing 25 mM glucose, 2 mM glutamine and 1 mM sodium pyruvate) and in response to 1.5 μM oligomycin, 1 μM FCCP, 1 μM rotenone and 4μM antimycin (Rot+Ant) with the Seahorse XF-96 Extracellular Flux Analyzer (Seahorse Bioscience). Real-time OCR was recorded according to the manufacturer’s manual. The software XFe Wave (Seahorse Bioscience) was utilized to examine the results.

### Macrophage preparation and polarization

PMs were harvested from BALB/c mice after intraperitoneal injection of 1 mL sterile 6% starch solution for 72 h. PMs were stimulated towards M1 or M2 polarization with lipopolysaccharide (LPS) (100 ng/mL, 24 h) to induce M1 or HCM (15%) to induce M2. The control group was stimulated with phosphate buffered saline.

### RNA extraction and qRT-PCR analysis

Total RNA was isolated from PMs using TRIzol reagent (Invitrogen) following the manufacturer’s instructions. qRT-PCR was performed using SYBR®-Green (Takara, Dalian, China) and an ABI Prism 7900 Sequence Detection System (Applied Biosystems, Foster City, CA, USA). The primers included CD206 forward, 5′-GGGACTCTGGATTGGACTCA-3′ and reverse, 5′-CCAGGCTCTGA TGATGGACT-3′; Arg-1 forward, 5′-CCCCAGTACCAACAGGACTACC-3′ and reverse, 5′-TGAACGTGGCGGAATTTTGT-3′; PPAR forward, 5′-TCCCATACACAACCGCAGTCGC-3′ and reverse, 5′-GGGGTCATTTGGTGACTCTGGGGT-3′; TNF-α forward, 5′-GGATCTCAAAGACAACCAAC-3′ and reverse, 5′-ACAGAGCAATGACTCCAAAG-3′; and GAPDH forward, 5′-CACCCACTCCTCCACCTTTG-3′ and reverse, 5′-CCACCACCCTGTTGCTGTAG-3′. Data were normalized to the expression of GAPDH.

### Western blot analysis

The cells were lysed using a cell lysis buffer (Cell Signaling, USA). A NE-PER nuclear protein extraction kit was used for nuclear protein extraction, following the manufacturer’s protocol (Thermo Scientific, USA).

### Immunohistofluorescence

Double immunofluorescence studies were carried out in frozen samples fixed in PFA. The primary antibodies were anti-iNOS (mouse monoclonal, 1:250, Santa Cruz Biotechnology) and anti-CD206 (goat polyclonal, 1:50, Santa Cruz Biotechnology), as well as anti-F4/80. The secondary antibodies included goat anti-rabbit (1:1000, Invitrogen), goat anti-mouse IgG Alexa Fluor 555 or 488 (1:1000, Invitrogen), and FITC-conjugated sheep anti-rat IgG (1:50, AbD Serotec).

### Analysis of cell proliferation and invasion

Hepa1–6 cells were cultured with the supernatant from SIRT4-knockdown M2 macrophages or controls. Cell Counting Kit-8 (CCK-8, Beyotime, China) was used for cell proliferation analysis. Cell invasion assays were performed in two parts of the studies. 1. In the study of SIRT4-knockdown M2 macrophages promoting invasion of HCC cells, 1 × 10^5^ Hepa1–6 cells were added to the upper chamber and co-cultured with the supernatant of SIRT4-knockdown M2 macrophages. 2. In the study of elevated MCP-1 expression promoting TAM infiltration, 1 × 10^5^ PMA-differentiated THP-1 cells were added to the upper chamber and co-cultured with the supernatant of SIRT4-knockdown HepG2 cells.

### IL-6, IL-10, and VEGF neutralization assay

HCM was used to stimulate shSIRT4-Lv and shCont-Lv-infected macrophages for 24 h. Then, supernatants were collected and preincubated with immunoglobulin G or the appropriate cytokine neutralization antibody before adding this mixture to Hepa1–6 cells. Three days later, the cells were analysed using CCK-8.

#### Statistical analysis

Data are reported as the mean values ± SEM. All experiments were performed in triplicate. Statistical significance was determined using SPSS 21.0 software. Student’s t test was used to assess the statistical significance of the differences between experimental groups, while the differences between groups were analysed by the log-rank test. A *p*-value < 0.05 was considered a significant difference.

## Results

### SIRT4 levels are significantly downregulated in HCC tumour tissues

**Fig. 1:**
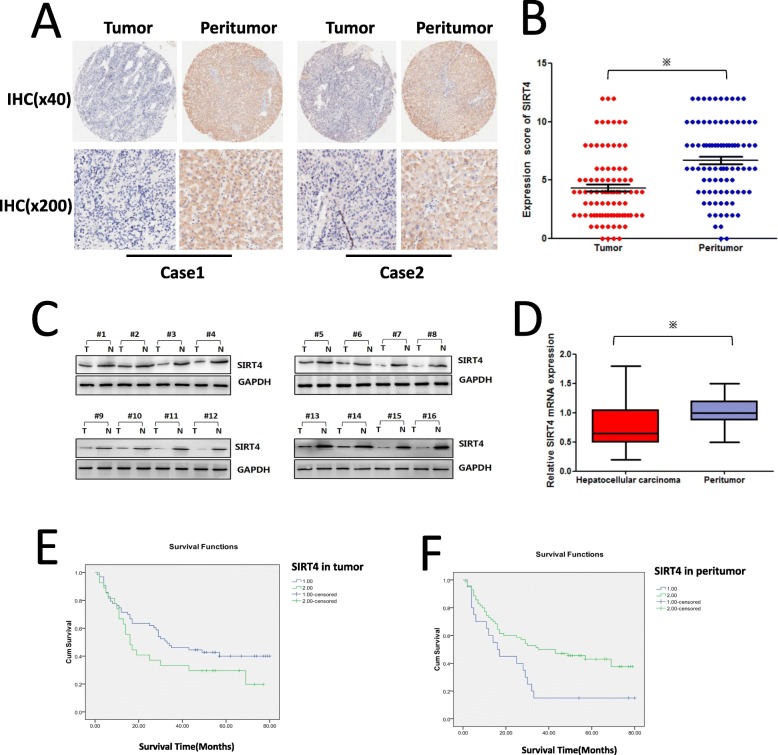
SIRT4 expression is downregulated in human HCC tissues, and the expression of SIRT4 in HCC peritumour tissues was positively associated with HCC survival. **a** Immunohistochemical staining was utilized to examine SIRT4 expression in HCC tumour tissues and matched peritumour tissues (magnification at × 40 and × 200). **b** Immunohistochemical scores of SIRT4 expression in HCC tumour tissues and peritumour tissues (* *P* < 0.05). **c** Western blot analysis of SIRT4 in HCC tissues and matched peritumour tissues. Equal protein loading was confirmed using GAPDH as a control. **d** The mRNA level of SIRT4 in HCC tissues and peritumour tissues (* *P* < 0.05). **e** The Kaplan–Meier survival curve showing the correlation between SIRT4 expression in tumour tissues and survival of HCC patients (*p* = 0.133), 1: low SIRT4 expression, 2: high SIRT4 expression. **f** The Kaplan–Meier survival curve demonstrated the correlation between SIRT4 expression in peritumour tissues and survival of HCC patients (*p* = 0.015), 1: low SIRT4 expression, 2: high SIRT4 expression

To elucidate the function of SIRT4 in HCC, tissue microarrays were used to examine the expression of SIRT4 in HCC tissues. Immuno-histochemical staining showed that SIRT4 was downregulated significantly in tumour tissues compared with matched surrounding tissues (Fig. 1a and b). The expression level of the SIRT4 protein in HCC tissues was significantly lower than that in peritumour tissues (1.233 ± 0.596 vs 1.922 ± 0.396, *P* = 0.000). Furthermore, total protein and RNA were extracted from fresh HCC tissues and matched peritumour tissues, and western blot and qRT-PCR assays confirmed that SIRT4 was downregulated in tumour tissues compared with peritumour tissues (Fig. 1c and d). The conclusion of these results is that SIRT4 expression is significantly downregulated in HCC tumour tissues.

### Downregulation of SIRT4 in HCC peritumour tissues is associated with poor survival of HCC patients

Correlation analysis between SIRT4 expression and the clinicopathological characteristics of HCC patients revealed that SIRT4 expression in HCC peritumour tissues was negatively associated with the tumour size (*r* = − 0.313, *p* = 0,003), pathological grade (*r* = − 0.266, *p* = 0.011), T stage (*r* = − 0.370, *p* = 0.001), and clinical stage (*r* = − 0.390, *p* = 0.000) of HCC patients (Table 1), but there was no correlation between SIRT4 expression in tumour tissues and clinicopathological characteristics of HCC patients (*p* > 0.05). Furthermore, as shown in Fig. 1e and f, Kaplan-Meier analysis and log-rank statistical test showed that SIRT4 levels in HCC peritumour tissues were positively associated with the survival of HCC patients (42.9% vs 15.0%, *p* = 0.015), while the expression of SIRT4 in tumour tissues was not associated with the prognosis of HCC patients (*p* = 0.133). Therefore, these results suggest that SIRT4 may play a tumour-suppressing role in HCC peritumour tissues by inhibiting the development and migration of tumour cells and thus improving the prognosis of patients.

**Table 1:** The correlation between SIRT4 expression and clinicopathological characteristics of HCC patients

			Gender	age	Tumor size	Differentiated stage	T	N	M	TNM stage
Spearman’s rho	Expression of SIRT4 in tumor tissues	Correlation	.024	−.055	.020	−.171	.089	.178	−.071	.056
		Coefficient								
		Sig. (2-tailed)	.821	.611	.855	.106	.428	.113	.523	.622
		N	90	89	89	90	82	81	82	81
	Expression of SIRT4 in para-carcinoma tissue	Correlation	.178	.057	−.313^a^	−.266^a^	−.370^a^	.058	−.210	−.390^a^
		Coefficient								
		Sig. (2-tailed)	.093	.596	.003	.011	.001	.609	.059	.000
		N	90	89	89	90	82	81	82	81

^a^Correlation is significant at the 0.05 level (2-tailed)

### Downregulation of SIRT4 is associated with increased macrophage infiltration and M2 macrophages in HCC peritumour tissues

**Fig. 2:**
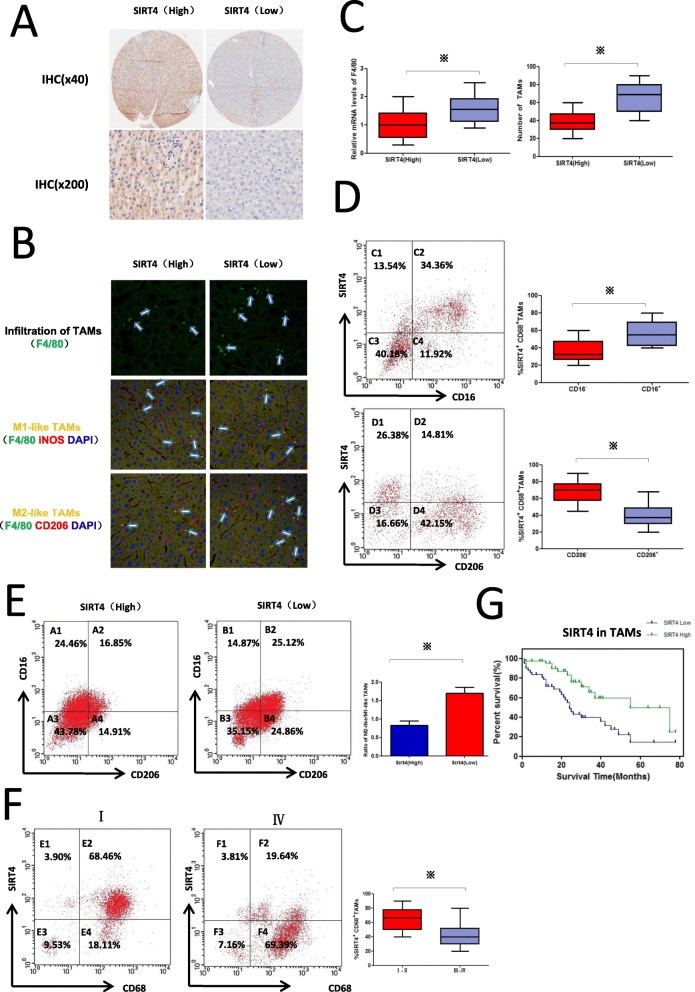
Downregulation of SIRT4 is associated with increased macrophage infiltration and M2 macrophages in HCC peritumour tissues. **a** Immunohistochemical staining was utilized to examine SIRT4 in HCC peritumour tissues (magnification at × 40 and × 200). **b** The cell phenotype in tissue sections from HCC patients was detected by F4/80+/iNOS+ for M1 and F4/80+/CD206+ for M2 using double immunohistofluorescence. The M1-like or M2-like macrophages are indicated by an arrow (400×). **c** QRT-PCR was used to detect F4/80 mRNA levels. Statistical analysis of the relationship between TAM infiltration and SIRT4 level. **d** FCM showed SIRT4 expression in CD206+ or CD206− TAMs and CD16+ or CD16− TAMs. **e** FCM analysis demonstrated TAM polarization in HCC peritumour tissues of different SIRT4 expression profiles. Statistical analysis between the ratio of M2/M1 macrophages and the SIRT4 level. **f** FCM analysis demonstrated SIRT4 expression in CD68+ TAMs from tumours with different grades. **g**. The Kaplan–Meier survival curve demonstrated the correlation between SIRT4 expression in CD68+ macrophages and survival of HCC patients. Data are displayed as the means ± SE, * *p* < 0.05

Our previous results suggest that SIRT4 expression in HCC peritumour tissues is associated with the clinicopathological characteristics and prognosis of patients. Therefore, the HCC cases were classified into two groups (SIRT4 High and SIRT4 Low) according to SIRT4 expression in HCC peritumour tissues as described in the Methods section (Fig. 2a). It has been reported that SIRT4 may affect the inflammatory response, so its role in the tumour immune microenvironment, especially in tumour-associated macrophages (TAMs) was investigated next. As shown in Fig. 2b and c, TAMs characterized by F4/80 expression were examined by immunohistofluorescence and qRT-PCR. We found that the number of TAMs or F4/80 expression was much higher in the SIRT4 Low group than in the SIRT4 High group. Furthermore, the phenotype of TAMs was characterized by double immunohistofluorescence, which involved co-staining with the macrophage marker F4/80 and either inducible nitric oxide synthase (iNOS) (M1 marker) or mannose receptor CD206 (M2 marker). As shown in Fig. 2b and e, immunohistofluorescence and FCM analysis both demonstrated that there was a higher ratio of M2/M1 macrophages in the SIRT4 Low group. These results demonstrated that there was a higher proportion of M2 polarized TAMs and increased TAM infiltration in the HCC peritumour tissues with low SIRT4 expression. SIRT4 may affect the polarization and infiltration of TAMs.

### SIRT4 is downregulated in M2-like TAMs and correlates with the poor survival of HCC patients

To investigate whether SIRT4 plays a role in TAM polarization, we investigated SIRT4 expression in the M1 and M2 distinct groups of CD68+ (macrophage marker) TAMs. As shown in Fig. 2d, FCM analysis showed that SIRT4 expression in CD16+ (M1 marker) TAMs was significantly higher than that in CD16- TAMs, and CD206+ (M2 marker) TAMs showed low SIRT4 expression. These results indicated that SIRT4 was mainly expressed in M1 TAMs and had a low expression level in M2 TAMs. Next, we further investigated SIRT4 expression in CD68+ macrophages from tumours with different grades. As shown in Fig. 2f, we found that SIRT4 expression in CD68+ TAMs from tumours with grades III–IV was much lower than that from tumours with grades I–II. Moreover, Kaplan-Meier analysis showed that the downregulation of SIRT4 in CD68+ macrophages was correlated with the poor survival of HCC patients (Fig. 2g).

### The HCC microenvironment inhibits SIRT4 expression in macrophages, and SIRT4 silencing facilitates M2 polarization

**Fig. 3:**
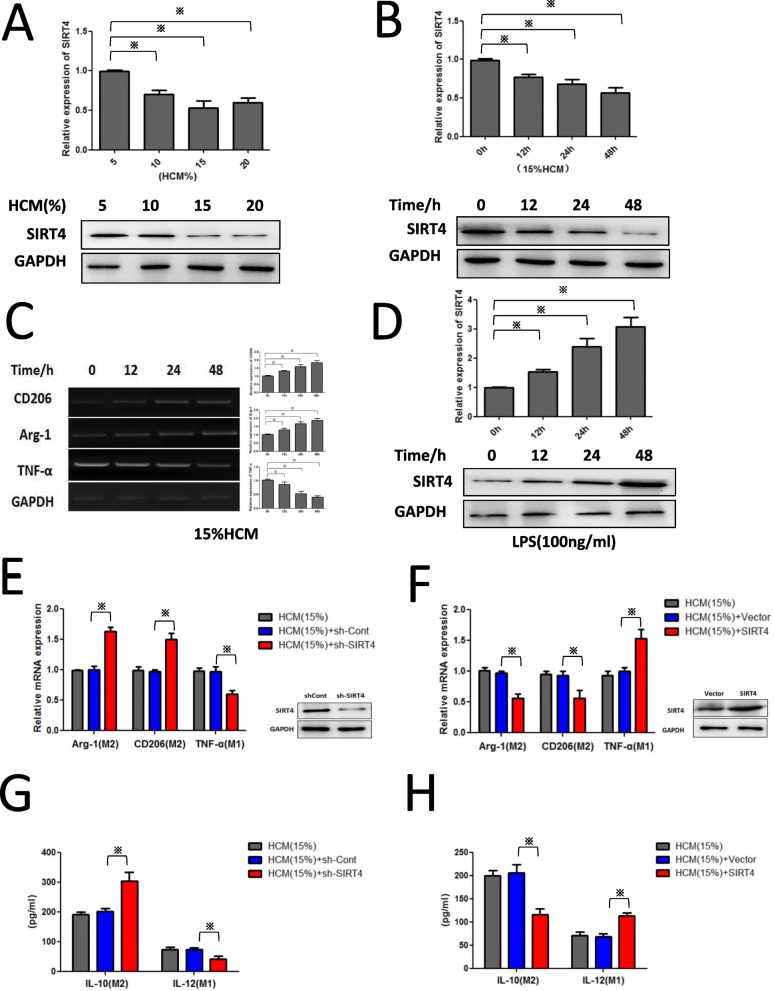
The HCC microenvironment inhibits SIRT4 expression in TAMs, and SIRT4 silencing facilitates M2-like polarization. **a**-**b** Western blotting and qRT-PCR were employed to detect the protein level and mRNA level of SIRT4 in PMs treated with HCM at various concentrations and time intervals. **c** PMs were treated with the indicated dose of HCM for 24 h and then qRT-PCR was performed to assess the expression of Arg-1, CD206, and TNF-α. **d** Western blotting and qRT-PCR were utilized to detect the protein level and mRNA level of SIRT4 in PMs treated with LPS (100 ng/ml) for different time intervals. **e**-**f** PMs were transfected with shSIRT4-Lv or SIRT4-Lv with the indicated dose of HCM for 24 h and qPCR was performed to evaluate the expression of Arg-1, CD206, and TNF-α. **g**-**h** An ELISA array was performed to evaluate the expression of IL-10 and IL-12. * *p* < 0.05

**Fig. 4:**
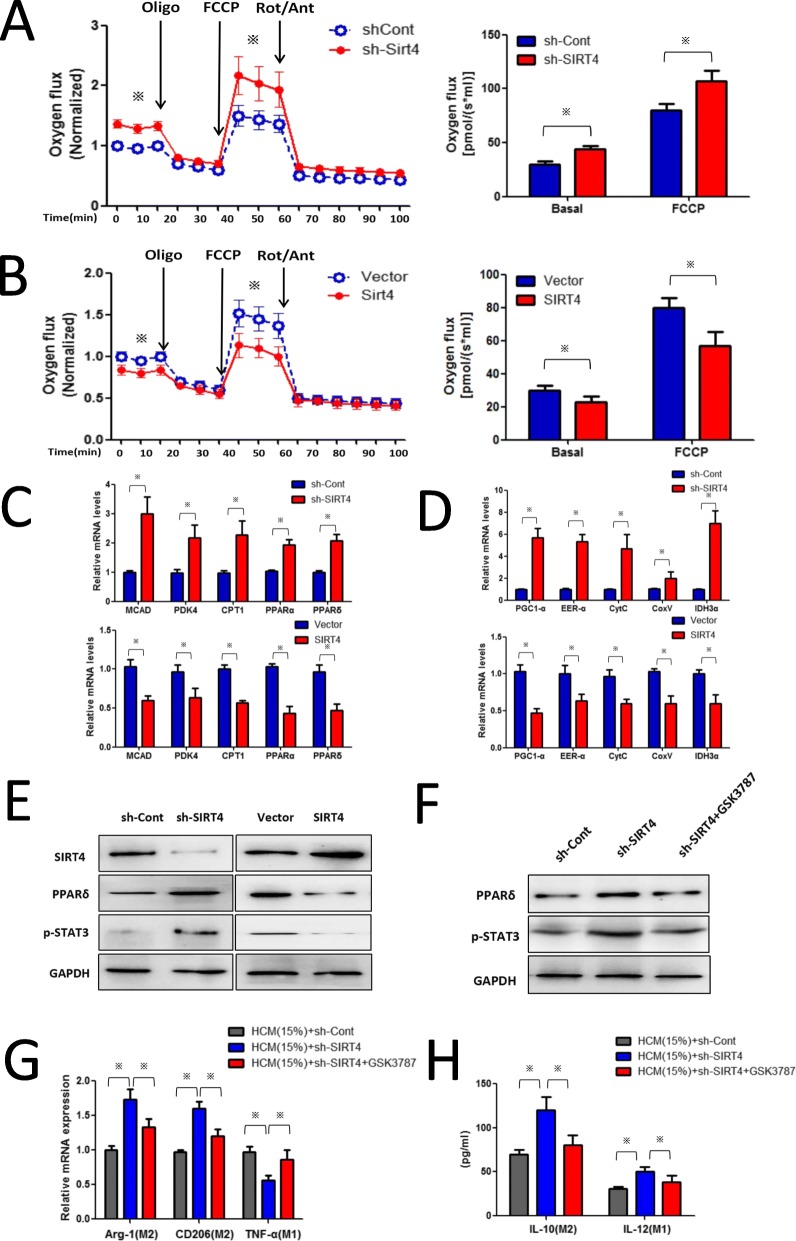
SIRT4 interference modulates TAMs to M2-like polarization via the FAO-PPARδ-STAT3 signalling pathway. **a** and **b** The oxygen flux (respiration) in control, SIRT4KD and SIRT4OE TAMs. The OCR was measured under basal and oligomycin, FCCP and Rotenone/Antimycin-A treatment conditions. **c** qRT-PCR was used to detect the mRNA level of FAO genes. **d** qRT-PCR was used to detect the mRNA level of the mitochondrial gene. **e** Western blotting was used to detect the effect of SIRT4 silencing on p-STAT3 nuclear translocation in TAMs. **f** Retrovirally knocked down PPARδ by its inhibitor GSK3787 reversed the effects of shSIRT4-Lv on p-STAT3 nuclear translocation. **g** Retrovirally knocked down PPARδ reversed the effects of shSIRT4-Lv on the levels of phenotype markers in TAMs. **h** Retrovirally knocked down PPARδ reversed the effects of shSIRT4-Lv on IL-10 and IL-12 expression in TAMs. Statistical significance was calculated using Student’s t-test: *p < 0.05

To test our hypothesis that SIRT4 may affect TAM polarization, HCM was used in experiments in vitro. As shown in Fig. 3a-c, HCM inhibited SIRT4 expression in PMs in a concentration- and time-dependent manner, and HCM-stimulated macrophages also displayed heightened expression of M2 markers (CD206, Arg-1) and reduced expression of an M1 marker (TNF-α). Furthermore, M1-like macrophages stimulated by LPS treatment displayed enhanced expression of SIRT4 (Fig. 4d). These results are consistent with our clinical results.

Due to the low SIRT4 expression in the M2 phenotype, we investigated the role of SIRT4 in alternative activation of macrophages. As shown in Fig. 3e and g, SIRT4 interference promoted M2 activation of HCM-stimulated macrophages (enhanced Arg-1, CD206, and IL-10 production but decreased TNF-α and IL-12 expression). However, the M2-like phenotype of TAMs could be reversed by overexpression of SIRT4 (Fig. 3f and h). These results indicated that SIRT4 affects the polarization of macrophages.

### SIRT4 silencing modulates TAM M2 polarization via the FAO-PPARδ-STAT3 signalling pathway

Lipid metabolism and its products play a key role in regulating macrophage functions in inflammation and resolution. It has been reported that M2 macrophage polarization is related to an increase in FA oxidation. Recent studies have revealed that SIRT4 is involved in lipid metabolism; therefore, we examined whether SIRT4 drives TAMs to M2-like polarization via an increase in FA oxidation. As shown in Fig. 4a, TAMs with SIRT4 silencing had increased oxygen consumption under basal and FCCP treatment conditions, whereas TAMs with SIRT4 overexpression (SIRT4OE) had decreased oxygen consumption (Fig. 4b). Next, we studied the lipid metabolic activities in SIRT4-knockdown TAMs by examining the expression of genes in the fatty-acid biosynthesis and fatty-acid oxidation pathways. As shown in Fig. 4c, we found that SIRT4 knockdown enhanced lipid catabolic gene expression in TAMs including MCAD (medium chain acyl-CoA dehydrogenase), PDK4 (pyruvate dehydrogenase kinase isoenzyme 4), CPT1 (carnitine palmitoyltransferase1), PPARδ, and PPARɑ. We further found that SIRT4 knockdown also increased mitochondrial gene expression in TAMs (Fig. 4d). Among these elevated lipid catabolic genes, PPARδ and the PPARδ-STAT3 axis have been reported to skew human macrophages to anti-inflammatory polarization. To investigate whether SIRT4 knockdown modulates TAM M2 polarization via the PPARδ-STAT3 axis by metabolic re-programming, the p-STAT3 protein level was examined. As shown in Fig. 4e, the p-STAT3 protein levels were enhanced by SIRT4 knockdown in TAMs. To further determine whether the PPARδ-STAT3 axis was responsible for M2 polarization of TAMs, we retrovirally knocked down PPARδ by its inhibitor GSK3787. As shown in Fig. 4f, PPARδ expression was successfully knocked down, and we found that the expression of p-STAT3 was restored to its lower level in SIRT4-knockdown TAMs. Moreover, PPARδ inhibition by GSK3787 restored the phenotype markers induced by SIRT4 knockdown in TAMs (Fig. 4g and h). These results indicated that SIRT4 modulates TAMs to M2-like polarization via the FAO-PPARδ-STAT3 signalling pathway.

### Silencing of SIRT4 in M2-like TAMs promotes the proliferation, migration, and invasion of HCC cells by enhancing IL-6 production

**Fig. 5:**
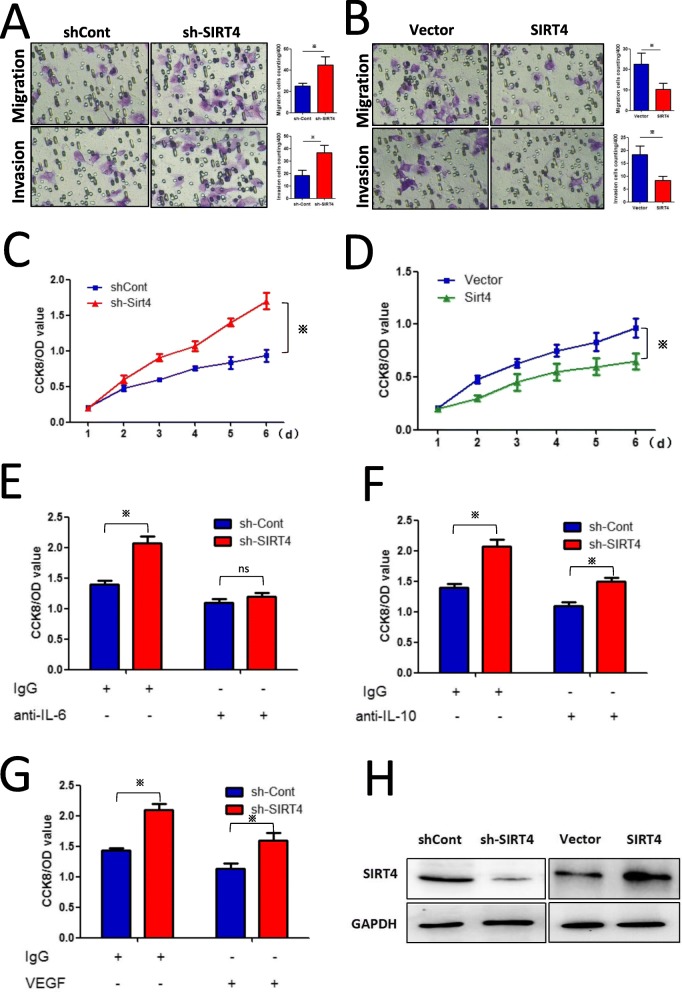
SIRT4 silencing in M2-like TAMs promotes the proliferation, migration, and invasion of HCC cells by enhancing IL-6 production. **a**-**b** Overexpression or silencing of SIRT4 in M2-like TAMs inhibited or promoted the migration and invasion of Hepa1–6 cells, respectively, as demonstrated by Transwell assays. **c**-**d** Overexpression or silencing of SIRT4 in M2-like TAMs inhibited or promoted the growth of co-cultured Hepa1–6 cells. **e** Hepa1–6 cells were treated with the supernatant (pre-incubated with IgG or anti-IL-6 neutralizing antibody) from shSIRT-Lv- or Ctr-Lv-infected M2 TAMs. Analysis of cell proliferation after 3 days using CCK-8 assay. **f**-**g** Hepa1–6 cells were treated with supernatant (preincubated with IgG or anti-IL-10 (VEGF) neutralizing antibody) from shSIRT-Lv- or Ctr-Lv-infected M2 TAMs. Analysis of cell proliferation after 3 days using CCK-8 assay. **h** Western blot analysis revealed that SIRT4 was successfully knocked down or overexpressed. *p < 0.05

Various studies have reported that M2-like TAMs promote HCC development and metastasis. As shown in Fig. 5a and b, Transwell assays confirmed that SIRT4 silencing in M2-like TAMs promoted the migration and invasion of Hepa1–6 cells in a co-culture system, and SIRT4 was successfully knocked down or overexpressed, as shown in Fig. 5h. Moreover, silencing of SIRT4 in M2-like TAMs promoted Hepa1–6 cell growth, and overexpression of SIRT4 significantly inhibited cell growth (Fig. 5c and d). ELISA results showed that SIRT4 silencing significantly enhanced the production of IL-6, IL-10 and VEGF, which are known to be the major pro-tumoural cytokines produced by TAMs. We found that neutralization of IL-6 critically decreased the proliferation of Hepa1–6 cells co-cultured with SIRT4-knockdown TAMs (Fig. 5e). However, neutralization of IL-10 or VEGF had little effect on shSIRT4-promoted HCC cell growth (Fig. 5f and g). These results indicated that SIRT4 interference promoted HCC cell growth by augmenting IL-6 production.

### SIRT4 silencing in M2-like TAMs promotes M1 macrophage apoptosis by enhancing IL-10 production in HCC peritumour tissues

**Fig. 6:**
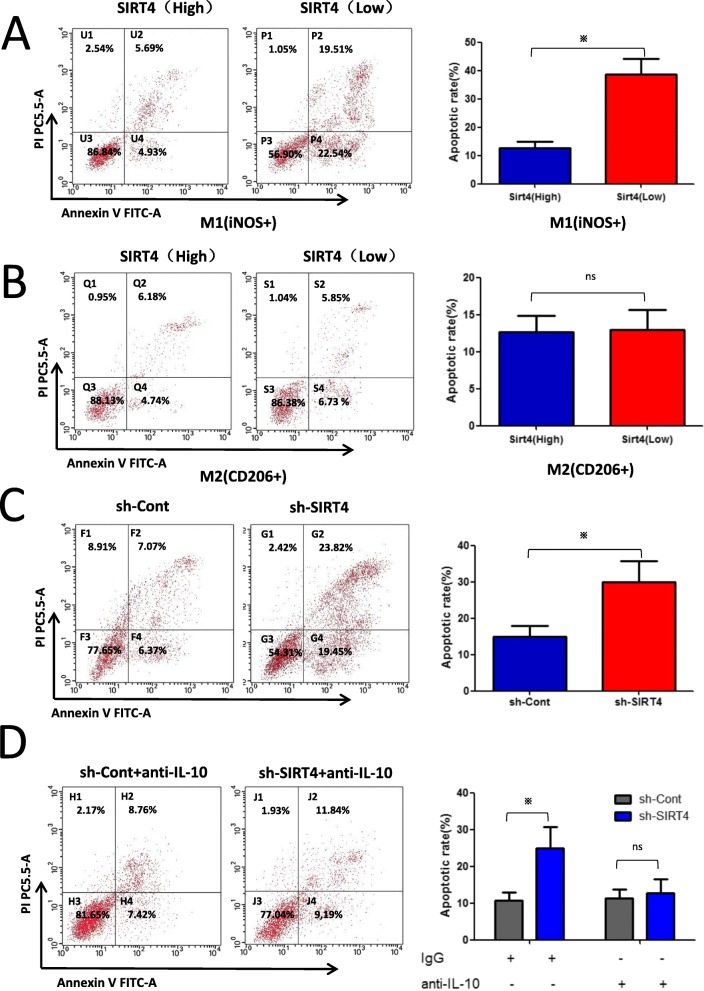
SIRT4 silencing in M2-like TAMs promotes M1 macrophage apoptosis by enhancing IL-10 production in HCC peritumour tissues. **a**-**b** Investigators characterized the phenotype of apoptotic TAMs (F4/80+) and the apoptotic rate of TAMs by flow cytometry using iNOS (M1) or CD206 (M2). **c** FCM showed the supernatant of SIRT4 interference in M2-like TAMs on M1 TAM apoptosis. The percentage of early apoptotic cells is located in the lower right quadrant (Annexin V-FITC-positive/PI-negative), and the percentage of late apoptotic cells is located in the upper right quadrant (Annexin V-FITC-positive/PI-positive). **d** Supernatant of SIRT4 knocked-down M2-like TAMs with the IL-10-neutralizing antibody removed the inhibiting effects of SIRT4 interference in M2-like TAMs on M1 TAM apoptosis. *p < 0.05

We previously found that there was a higher ratio of M2/M1 macrophages in HCC peritumour tissues with low SIRT4 expression. In addition, as shown in Fig. 6a, we found that the apoptotic rate of M1-like TAMs was higher in HCC peritumour tissues with low SIRT4 expression. However, as shown in Fig. 6b, the apoptotic rate of M2-like TAMs was not significantly different. We then aimed to determine the cause of the increase in M1 TAM apoptosis. As previously reported [15], IL-10 secreted by M2 Kupffer cells induced M1 apoptosis through the stimulation of arginase in high iNOS-expressing M1 KCs. Therefore, we investigated the effect of SIRT4 silencing in M2-like TAMs on M1 TAM apoptosis via FCM. We found that the supernatant of SIRT4-knockdown M2-like TAMs significantly promoted M1 TAM apoptosis (Fig. 6c), and the anti-IL-10 neutralizing antibody weakened the effects of SIRT4-knockdown M2-like TAMs on M1 TAM apoptosis (Fig. 6d). These results clearly revealed that increased M2 polarization of TAMs and apoptosis of M1 TAMs were responsible for the higher ratio of M2/M1 TAMs in HCC peritumour tissues.

### SIRT4 silencing in M2-like TAMs promotes the development of H22 homografts in BALB/c mice

**Fig. 7:**
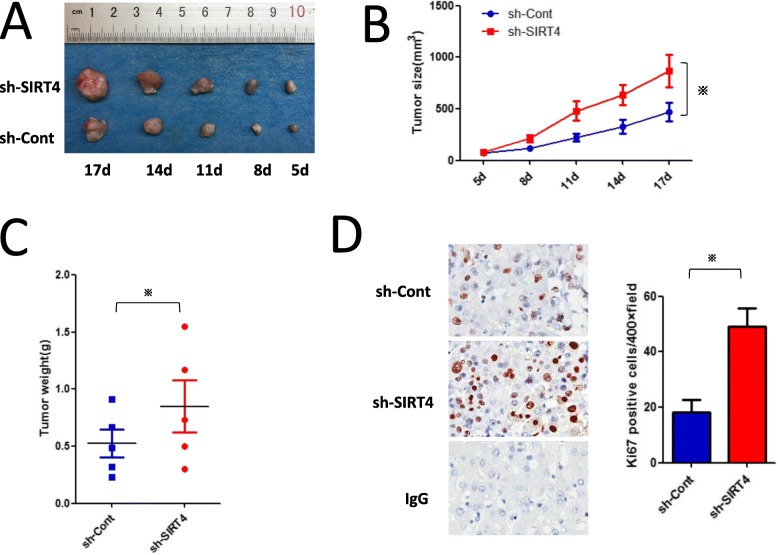
SIRT4 interference in M2-like TAMs promotes H22 homograft development in BALB/c mice. **a** BALB/c mice were subcutaneously injected with shSIRT4-Lv-infected M2 (Ctr-Lv as control) along with H22 cells. Tumour weights are represented. Images are provided from each group. **b** The tumour size served as the measurement for H22 homograft development. **c** Summary data for each group are provided (*n* = 5). **d** Immunohistochemical staining of Ki67 in tumour sections (left). Summary data are shown on the right. *p < 0.05

To determine the effects of SIRT4 silencing in M2-like TAMs on the progression of HCC in mice, we established a subcutaneous tumour model as previously described in the Methods section. In comparison to the controls, SIRT4 silencing in M2-like TAMs significantly promoted the development of H22 homografts (Fig. 7a and b). Similarly, tumour weight in the control TAM group was less than that in the SIRT4-knockdown M2-like TAM group (Fig. 7c). Ki67 immunochemical staining of shSIRT4-Lv-treated tumour section was markedly increased over that of the controls (Fig. 7d). Our data indicate that SIRT4 silencing in M2-like TAMs stimulates HCC growth in vivo.

### Elevated MCP-1 expression is responsible for increased TAM infiltration in HCC peritumour tissues

**Fig. 8:**
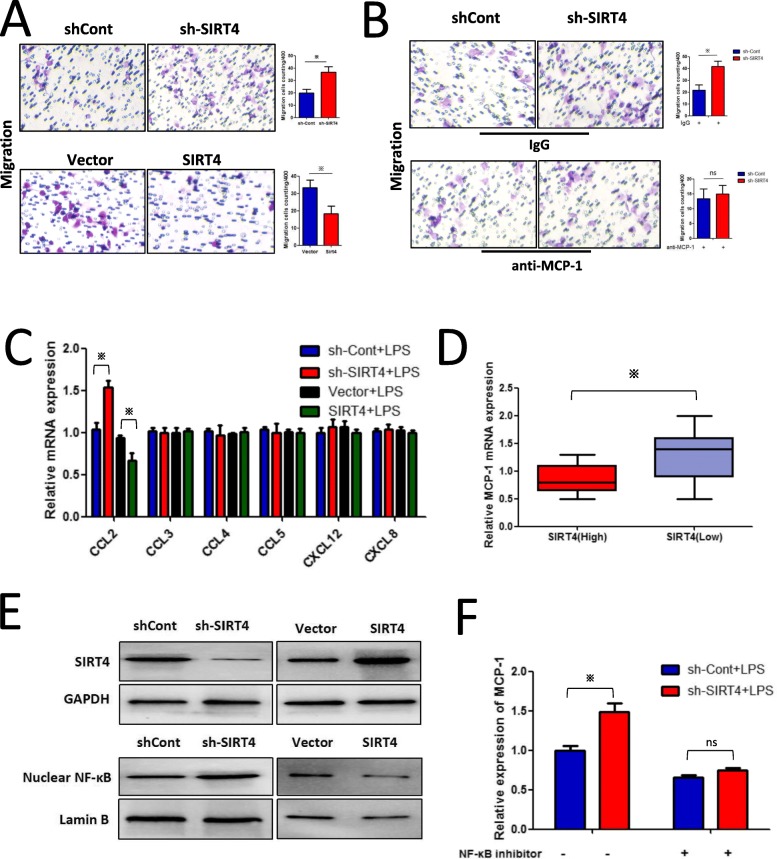
Elevated MCP-1 expression induced by SIRT4 silencing is responsible for increased TAM infiltration in HCC peritumour tissues. **a** The supernatant of SIRT4-knockdown or overexpression HepG2 cells promoted or inhibited the migration of co-cultured THP-1 cells, respectively. **b** The supernatant of SIRT4-knockdown HepG2 cells with the anti-MCP-1 neutralizing antibody reversed the effects of HepG2 cells on co-cultured THP-1 cells. **c** The mRNA expression levels of cytokines were determined by qRT-PCR. **d** Elevated expression of MCP-1 was confirmed in HCC peritumour tissues with low SIRT4 expression. **e** Effects of SIRT4 on p65 nuclear translocation. Cell nuclei were isolated for western blot analysis. **f** Suppression of NF-κB induced by its inhibitory ligand reversed the effects of shSIRT4-Lv on MCP-1 expression. * *p* < 0.05

Previously, we found that downregulation of SIRT4 was associated with increased macrophage infiltration in HCC peritumour tissues. Therefore, we established a co-culture system of HepG2 cells and PMA-differentiated THP-1 cells in Transwell chambers to study the interactions between the two cell types. As shown in Fig. 8a, the Transwell assays confirmed that SIRT4 silencing in HepG2 cells significantly increased the migration of PMA-differentiated THP-1 cells. As macrophage infiltration may be due to several monocyte/macrophage chemo-attracting agents, we examined the effect of SIRT4 knockdown on the expression of several monocytes/macrophage chemo-attracting agents (CCL2, CCL3, CCL4, CCL5, CXCL12, and CXCL8) in HepG2 cells. MCP-1, also called CCL2, is a key chemokine controlling macrophage accumulation and penetration. As shown in Fig. 8c, SIRT4 knockdown significantly upregulated monocyte chemotactic protein-1 (MCP-1) expression, which was further confirmed in HCC peritumour tissues (Fig. 8d). Neutralization of MCP-1 critically weakened the effects of SIRT4 silencing in HepG2 cells on the migration of PMA-differentiated THP-1 cells (Fig. 8b).

**Fig. 9:**
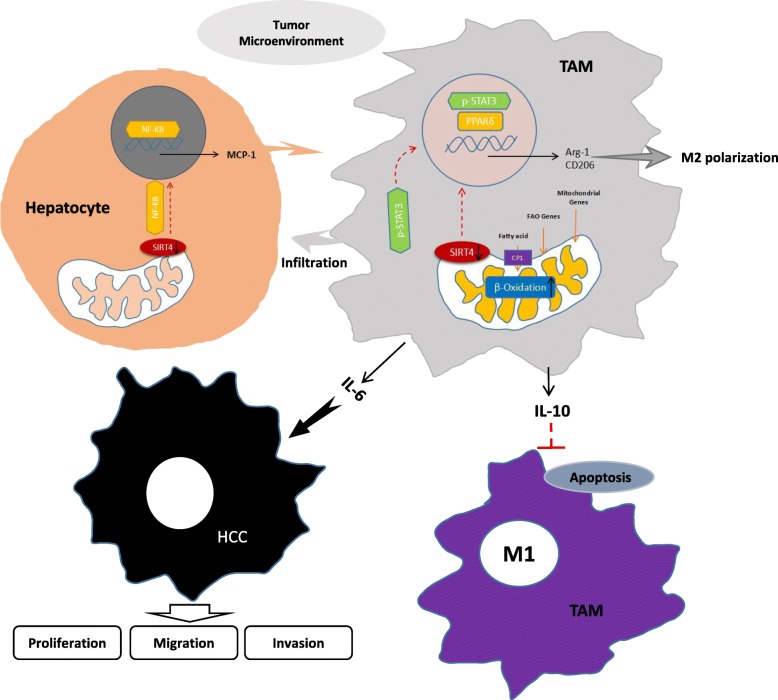
The HCC microenvironment inhibits SIRT4 expression in TAMs and modulates alternative activation of TAMs, which contributes to HCC development. The HCC microenvironment inhibits SIRT4 expression in TAMs. The downregulation of SIRT4 increases FA oxidation and the expression of lipid catabolic genes, which induces alternative activation of TAMs via the FAO-PPARδ-STAT3 signalling pathway. Alternatively activated TAMs produce IL-6 to accelerate HCC development and produce IL-10 to accelerate M1 macrophage apoptosis. On the other hand, downregulation of SIRT4 in hepatocytes activates MCP-1 expression to increase TAM infiltration, which accelerates HCC development via the NF-κB signalling pathway

As the transcription factor NF-κB is a well-known transcription controller of MCP-1, we explored the NF-κB-MCP-1 axis in HepG2 cells. We found that SIRT4 silencing aggravated p65 nuclear translocation in HepG2 cells (Fig. 8e), whereas inhibition of NF-κB by its inhibitor ammonium pyrrolidinedithiocarbamate (PDTC) reversed the effects of shSirt4-Lv on MCP-1 cells (Fig. 8f), supporting our hypothesis that SIRT4 inhibited MCP-1 expression via the NF-κB pathway in HCC peritumour tissues.

## Discussion

SIRT4 is a mitochondrial sirtuin that plays the role of an efficient ADP-ribosyltransferase but weak protein deacetylase dependent on stringent substrate specificity [17, 18]. It has been reported that SIRT4 regulates fatty acid oxidation and mitochondrial gene expression in liver and muscle cells [19]. In addition, previous studies have demonstrated that SIRT4 has protective properties through anti-apoptosis activity [20]. Importantly, SIRT4 can regulate insulin secretion and has tumour suppressor activity via modulation of glutamate dehydrogenase [16, 21]. SIRT4 also plays a role in the inflammatory response in some tissues [22]. However, the role of SIRT4 in the development of HCC is still unclear.

This is a study to report a relationship between SIRT4 expression and prognosis in hepatocellular carcinoma. We selected HCC tumour tissues and matched peritumour tissues from 90 patients who were followed for 4–7 years to create an HCC tumour tissue microarray for immunohistochemical staining analysis. TMA and IHC results suggested that the expression of SIRT4 in HCC tumour tissue was significantly decreased compared to that in the matched peritumour tissues. Through Kaplan-Meier survival analysis and the log-rank statistical test for single-factor analysis of survival, we found that SIRT4 expression in the peritumour tissues was positively correlated with the survival of HCC patients, and there was no correlation between the expression of SIRT4 in HCC tumour tissues and pathological characteristics (*P* > 0.05). After NPar pairing analysis and Spearman’s tests, we found that SIRT4 expression in peritumour tissues was negatively associated with the tumour size, pathological grade, T stage, and clinical stage of HCC patients (*r* = − 0.313, *p* = 0,003; *r* = − 0.266, *p* = 0.011; *r* = − 0.370, *p* = 0.001; and *r* = − 0.390, *p* = 0.000, respectively). It seems that SIRT4 plays a tumour-suppressing role mainly in peritumour tissues. Since metastasis recurrence after radical operation occurs mostly in the postoperative residual liver, the microenvironment of the residual liver, especially the inflammatory microenvironment, can directly affect the prognosis of postoperative HCC patients. Therefore, we classified all HCC cases into two groups (SIRT4 High and SIRT4 Low) according to SIRT4 expression in HCC peritumour tissues. Using immunohistofluorescence and FCM analysis, we found two interesting phenomena: a higher ratio of M2/M1 macrophages and increased macrophage infiltration in HCC peritumour tissues.

It has been reported that a high density of tumour-infiltrating macrophages can predict poor prognosis in post-surgical HCC patients [23]. Here, our data supported this view. In this study, we found that elevated MCP-1 expression induced by the downregulation of SIRT4 in HCC peritumour tissues was responsible for the increased TAM infiltration. A key consideration is the mechanism for the upregulation of MCP-1 in HCC peritumour tissues. In subsequent experiments, we found that downregulation of SIRT4 could activate the NF-κB pathway, resulting in the downstream upregulation of MCP-1 gene expression. It has been shown that various members of the NF-κB/IKK signalling pathway are purportedly found in mitochondria [24]. Some examples include the NF-κB subunits RelA and p50, the IκBɑ inhibitor and the upstream kinases IKKɑ, IKKβ, and IKKɣ [25]. It was found that p50 NF-κB and p65 NF-κB could be poly-adenosine diphosphate (ADP) ribosylated through interaction with PAR polymerase 1 (PARP1) [26]. Our hypothesis is that SIRT4 may have the ability to catalyse the ADP ribosylation of NF-κB in mitochondria, thereby increasing p65 nuclear translocation. We will continue to explore the detailed mechanisms in future experiments.

The other observed phenomenon was the increased ratio of M2/M1 macrophages in HCC peritumour tissues. It is well known that TAMs are important elements of the tumour microenvironment, and their alternative activation critically affects the growth of HCC [27]. Previous studies have also reported that SIRT4 can re-programme endotoxin tolerance and promote acute inflammation resolution in monocytes [28]. In this study, HCM inhibited SIRT4 expression, and SIRT4 silencing stimulated macrophages towards M2 polarization. Lipid metabolism and its products play an important role in modifying macrophage functions in terms of inflammation and resolution [29]. Macrophage M2 polarization is associated with the activation of oxidative metabolism, which includes a functional tricarboxylic acid (TCA) cycle fuelled by glutamine and glucose catabolism, an increase in mitochondrial oxidative phosphorylation, and the enhancement of FA oxidation (FAO) [30]. It has been demonstrated that macrophage M2 polarization induced by IL-4 requires a peroxisome proliferator-activated receptor gamma (PPARɣ), which is a nuclear receptor activated by FA derivatives [31]. Treatment of macrophages with IL-4 induces the expression of genes involved in FA uptake and increased FA oxidation [32]. Mechanistically, this process involves the phosphorylation of signal transducer and activator of transcription 6 (STAT6) and PPARɣ co-activator 1 beta (PGC1-β). Similar to PPARɣ, PPARδ also appears to be an important regulator of alternative activation of resident macrophages [33, 34]. Previous studies have reported that PPARδ regulates arginase I expression and the immunologic phenotype in alternatively activated Kupffer cells and that PPARδ regulates Kupffer cell alternative activation by re-programming the lipid metabolism [35]. A more recent study revealed that SIRT4 is involved in lipid metabolism. Therefore, we examined whether SIRT4 modulates TAMs to M2-like polarization via an increase in FA oxidation. We found that FAO genes including MCAD, PDK4, CPT1, PGC1-ɑ, PPARδ, and PPARɑ were increased in SIRT4-knockdown TAMs, and p-STAT3, which is essential for differentiation into the M2 phenotype, was also increased. Our data indicate that SIRT4 modulates TAMs to M2-like polarization via the FAO-PPARδ-STAT3 signalling pathway.

On the other hand, the energy sensor AMP-activated protein kinase (AMPK) may also be a significant mechanism of FAO regulation in macrophages [36]. AMPK is able to activate FAO at the expense of FA synthesis through at least two different mechanisms: first, phosphorylation and inactivation of acetyl-CoA carboxylase (ACC) (the first step of FA synthesis) and second, activation of PGC1-ɑ, which in turn stimulates mitochondrial biogenesis and mitochondrial function. Because SIRT4 can also affect the AMPK pathway, we will explore the relationship between the M2 polarization of TAMs and the SIRT4-AMPK-FAO axis in future studies.

In addition, SIRT4 silencing in HCM-stimulated TAMs significantly promoted the production of cytokines including IL-10, IL-6 and VEGF, which have been reported to promote tumour progression, and SIRT4 silencing inhibited IL-12 production, enhancing antitumour immunity and further preventing the development of cancer. Thus, SIRT4 silencing in macrophages significantly promoted macrophage-induced tumour development both in vitro and in vivo. We also focused on how SIRT4 inhibited TAM-mediated HCC progression. This study may point to the important effects of SIRT4 on the STAT3-IL-6 axis, which will be determined in our future experiments.

Studies have reported that tumour cells can “educate” macrophages in different areas [37]. We know that human tumour tissues can be categorized into the cancer nest area, invading edge, and peritumoural stroma. Depending on different microenvironments, macrophages acquire specific phenotypes with distinct functions [38, 39]. Cancer cells produce elements that corrupt the development of surrounding macrophages, briefly triggering the untimely activation of monocytes (M1-like) in the peritumoural region. This provokes the development of suppressive macrophages (M2-like) in the cancer nests, overtaking the inflammatory response [40, 41]. However, the mechanism of the M1 to M2 transition is not clear. One hypothesis is that M2-like macrophages might “educate” M1-like macrophages towards apoptosis. In our study, we found that the apoptotic rate of M1-like TAMs was higher in HCC peritumour tissues with low SIRT4 expression. We found that high IL-10 expression induced by SIRT4 silencing in M2-like TAMs might promote M1 macrophage apoptosis [15]. This highlights the hypothesis that SIRT4 might engage in the dynamic education of macrophages in HCC. In addition, it has been reported that a high ratio of M2/M1 macrophages in peritumour tissues is associated with a poor prognosis in patients after resection [42]. The results of this study may provide a powerful explanation for this observation.

## Conclusion

This study suggested that SIRT4 was significantly downregulated in HCC tumour tissues and that the expression of SIRT4 in HCC peritumour tissues was positively associated with HCC survival. The results from clinical studies demonstrated that downregulation of SIRT4 was associated with increased macrophage infiltration and M2 macrophages in HCC peritumour tissues. The reason for this is that downregulation of SIRT4 in TAMs modulates the alternative activation of macrophages and promotes HCC development via the FAO-PPARδ-STAT3 axis. On the other hand, elevated MCP-1 expression induced by downregulation of SIRT4 in HCC peritumour tissues is responsible for increased TAM infiltration. This study indicates that SIRT4 plays a role in HCC progression and may offer an important area of research for a new HCC treatment strategy (Fig. 9).

## Supplementary information

**Additional file 1:** **Table S1.** Clinicopathological Characteristics of Patients with Hepatocellular Carcinoma (*n*=90).

## Data Availability

All data generated during this study are included in this published article. [and its supplementary information files].

## Abbreviations

FAO: Fatty acid oxidation
FCM: Flow cytometry
HCC: Hepatocellular carcinoma
HCM: HCC-conditioned medium
MCP-1: Monocyte chemotactic protein-1
PCR: Polymerase chain reaction
SIRT4: Sirtuin 4
TAMs: Tumour-associated macrophages
