# Supplementary material for: SIRT4 silencing in tumor-associated macrophages promotes HCC development via PPARδ signalling-mediated alternative activation of macrophages
Source: J Exp Clin Cancer Res. 2019 Nov 19;38:469. doi: 10.1186/s13046-019-1456-9 (PMC6862746; doi:10.1186/s13046-019-1456-9)
Supplement: Supplementary file 1 — Additional file 1: Table S1. Clinicopathological Characteristics of Patients with Hepatocellular Carcinoma (n=90). [file 13046_2019_1456_MOESM1_ESM.docx]

**Additional file 1: Table 1. Clinicopathological Characteristics of Patients with Hepatocellular Carcinoma (n=90).**

| **Characteristics** | | **Value** | **%** |
| --- | --- | --- | --- |
| **Sex** | | | |
| **Male** | | **80** | **88.88** |
| **Female** | | **10** | **11.11** |
| **Age** | | | |
| **median(range), years** | **55(27~78)** | | |
| **Pathological grading** | | | |
| **I** | | **2** | **2.22** |
| **I~II** | | **4** | **4.44** |
| **II** | | **41** | **45.56** |
| **II~III** | | **17** | **18.89** |
| **III** | | **26** | **28.89** |
| **Toumor size** | | | |
| **Range, cm** | **1*11*1~30*15*10** | | |
| **TNM** | | | |
| **T1** | | **10** | **11.11** |
| **T2** | | **30** | **33.33** |
| **T3** | | **39** | **43.33** |
| **T4** | | **5** | **5.56** |
| **Unknown** | | **6** | **6.67** |
| **N0** | | **83** | **92.22** |
| **N1** | | **1** | **1.11** |
| **Nx** | | **1** | **1.11** |
| **Unknown** | | **5** | **5.56** |
| **M0** | | **84** | **93.33** |
| **M1** | | **2** | **2.22** |
| **Mx** | | **1** | **1.11** |
| **Unknown** | | **3** | **3.33** |
| **AJCC clinical stage** | | | |
| **I** | | **10** | **11.11** |
| **II** | | **30** | **33.33** |
| **III** | | **39** | **43.33** |
| **III-IV** | | **1** | **1.11** |
| **IVA** | | **1** | **1.11** |
| **IVB** | | **1** | **1.11** |
| **Unknown** | | **8** | **8.89** |
| **Suvival status** | | | |
| **Alive** | | **31** | **34.44** |
| **Dead** | | **59** | **65.56** |
